# Role of heparin-induced HGF release in the acute phase of STEMI

**DOI:** 10.3389/fcvm.2025.1668882

**Published:** 2025-11-06

**Authors:** S. Leboube, A. Paccalet, C. Brun, F. Moulin, B. Pillot, G. Bidaux, L. Mechtouff, H. Thibault, T. Bochaton, C. Crola Da Silva

**Affiliations:** 1 Institut de Cardiologie, Services d’Explorations Fonctionnelles Cardiovasculaires, Hospices Civils de Lyon, Bron, France; 2 CarMeN Laboratory, INSERM, INRAE, Universite Claude Bernard Lyon 1, Bron, France; 3 Stroke Department, Hospices Civils de Lyon, Bron, France

**Keywords:** hepatocyte growth factor (HGF), STEMI (myocardial infarction), heparin, cardioprotection, Met

## Abstract

**Introduction:**

Reperfusion injury remains a major limitation in the management of ST-segment elevation myocardial infarction (STEMI). Despite numerous preclinical successes, cardioprotective strategies have largely failed in clinical translation. Heparin, routinely administered for STEMI, may exert protective effects beyond anticoagulation through a rapid release of hepatocyte growth factor (HGF), a known cardioprotective agent.

**Methods:**

In this study, we analyzed 229 STEMI patients undergoing primary percutaneous coronary intervention from the HIBISCUS-STEMI cohort. Serum HGF levels were measured by using ELISA at five time points post admission. In a subset of four patients, HGF levels were assessed before and 30 min after heparin injection. To test the functional effect of HGF, a murine ischemia-reperfusion model was used where recombinant HGF (0.3 mg/kg) or saline was administered intravenously five minutes before reperfusion. Infarct size was quantified by 2,3,5-triphenyltetrazolium chloride staining.

**Results:**

A rapid and significant rise in HGF levels was observed at admission (median 8,750 pg/mL), declining thereafter. In the subset analysis, heparin administration increased HGF levels from 356 ± 77 to 5,026 ± 1,957 pg/mL (*p* < 0.05). In mice, HGF administration significantly reduced the infarct size compared with controls (48% vs. 58%, *p* = 0.0023), with no difference in the area at risk.

**Discussion:**

This study demonstrates that heparin induces a rapid and substantial increase in circulating HGF in STEMI patients, potentially mediating cardioprotection during reperfusion. These findings suggest that comedications like heparin may confound cardioprotective trials and should be considered in future translational strategies.

## Introduction

Developing a treatment limiting myocardial reperfusion injury remains a challenge. Different strategies targeting intracellular pathways, cardiomyocytes, non-cardiomyocyte cells, circulating cells, or microvascular obstruction have yielded strong preclinical results; however, their translation to the clinic has been unsuccessful (1). Heparin is an anticoagulant, established as the standard of care in ST-segment elevation myocardial infarction (STEMI) patients (2). But heparin has other properties that may play a role in cardioprotection. Because of its chemical similarities with connective tissue glycosaminoglycans such as heparan sulfate, heparin can bind with quiescent growth factors (3), especially hepatocyte growth factor (HGF), known as a target for cardioprotection (4–6). Salbach et al. showed that intravenous injection of heparin in humans or rats causes a massive and extremely rapid release of HGF into the blood (7). In STEMI patients, heparin is injected a few minutes before myocardial reperfusion. Therefore, patients are injected with HGF during reperfusion. The recent literature highlights the importance of comedications as confounders in cardioprotection. In this study, we hypothesize that heparin-induced HGF release protects the heart against myocardial ischemia-reperfusion injury, thus interfering with current cardioprotective strategies. This protection could partly explain the frustrating results observed in human cardioprotective strategies.

## Methods

### Hibiscus—STEMI cohort

The study cohort constituted patients admitted to Hospices Civils de Lyon with a diagnosis of acute STEMI during the period between 2016 and 2019. The study was approved by our institutional review board and Ethics Committee, and was registered on ClinicalTrials.gov (NCT03070496). All patients underwent a coronary angiogram at admission with reperfusion through primary percutaneous intervention (PCI).

Among the 229 patients included in the study, data on anticoagulant use were available for 226 individuals. Within this subgroup, 132 patients (58.41%) received unfractionated heparin (UFH) and 94 patients (41.59%) received low-molecular-weight heparin (LMWH). UFH was administered at a dose of 50 IU/kg IV, consistent with current recommendations in the context of primary PCI. LMWH (typically enoxaparin) was used according to standard dosing protocols, including a 30 mg IV bolus followed by 1 mg/kg SC, with dose adjustments based on patient age. No differences were observed at the peak of HGF between LMWH and UFH (respectively, 8,898 and 8,723 pg/mL, *p* = 0.77). Anti-Xa and aPTT levels were not systematically measured in this cohort.

### HGF measurements by the ELISA method

Sera were collected at five time points: Admission, 4 h after revascularization (H4), 24 h (H24), 48 h (H48), and 1 month after MI. HGF levels in the sera were determined using enzyme-linked immunosorbent assay (ELISA) (DuoSet DY294, R&D Systems, sensitivity range = 125–8,000 pg/mL) according to the manufacturer's instructions. All samples were measured simultaneously to avoid the interassay coefficient of variation. Blood samples were thawed only once.

### Animals

All procedures followed the principles and guidelines established by the European Convention for the Protection of Laboratory Animals and were approved by the Lyon 1 Claude Bernard University Committee for Animal Research. All experiments were performed in C57/BL6 male mice. Animals were housed in stable groups of four in individually ventilated cages with standard nesting materials and *ad libitum* access to filtered water and standard diet.

Mice were anesthetized with subcutaneous buprenorphine (0.075 mg/kg), followed by intraperitoneal alfaxalone and medetomidine. Local lidocaine infiltration was applied prior to surgery. Animals were orally intubated and mechanically ventilated, with continuous monitoring and adjustment of respiratory parameters and body temperature (37 °C ± 0.5 °C).

Ischemia was realized by ligation of the anterior interventricular artery, maintained for 60 min, followed by 24 h of reperfusion. Treatments were administered intravenously at 5 min before reperfusion [physiological saline (0.9% NaCl) or recombinant Mouse HGF Protein (2207-HG-025/CF, R&D Systems) at 0.3 mg/kg was injected via the tail vein]. In experimental animal models, no toxicity has been reported following intravenous administration of HGF (4). Animals were extubated upon awakening and monitored postoperatively to minimize pain.

Randomization was performed using simple randomization via Excel, with investigators blinded during surgery and analysis.

Infarct size was determined by colorimetric staining. Unisperse Blue pigment was injected to delineate the perfused and at-risk areas. The infarct size was measured using 2,3,5-triphenyltetrazolium chloride (TTC), which allows the identification of the necrosis area by planimetry. The infarct size was calculated as a ratio of the area of necrosis to the area at risk and expressed as a percentage.

### Statistics

A Shapiro–Wilk test was performed to assess the normality of continuous variables. Depending on the distribution, data were presented as mean ± standard deviation for normally distributed variables or as median (interquartile range) for non-normally distributed variables. Group comparisons were performed using parametric tests (e.g., Student's *t*-test) or non-parametric tests (e.g., Mann–Whitney *U* test), as appropriate, based on the distribution of the data assessed by using the Shapiro–Wilk test. We compared the normalized infarct size between the control and the recombinant HGF groups using a linear regression model with the group as a categorical predictor. A *p*-value < 0.05 was considered significant. Statistical analyses were performed using GraphPad Prism 8.01.

## Results

In the present analysis, 229 patients were included. The mean age of the patients was 59 ± 12 years, of whom 79.5% were male. The patients presented multiple risk factors with current smoking habit (59.7%), diabetes mellitus (14.8%), and hypertension (28.8%). The location of acute myocardial infarction was anterior in 51.5% of patients and 86% belonged to Killip class I at admission. Overall, 158 patients (69%) had a TIMI 0 coronary flow on invasive angiography (Table 1).

**Table 1:** Baseline characteristics of the study population (*N* = 229).

Patients characteristics	*n* = 229
Age, years ± SD	59 ± 12
Male sex, *n* (%)	182 (79.4)
Body mass index (BMI), kg/m^2^ (IQR)	26.7 (23.9–29.4)
Hypertension, *n* (%)	66 (28.8)
Hypercholesterolemia, *n* (%)	66 (28.8)
Diabetes mellitus, *n* (%)	34 (14.8)
Current smoking, *n* (%)	116 (50.7)
Clinical characteristics	
Time from symptom to PCI (min)	195 (137–290)
Anterior MI, *n* (%)	118 (51.5)
Killip status = 1, *n* (%)	197 (86)
TIMI at admission = 0, *n* (%)	158 (69)
Biochemical analyses	
Peak troponin I, ng/L (IQR)	42,792 (12,236–110,323)
Peak creatine Kinase Peak, mUI/L (IQR)	1,563 (600–3,685)
Peak CRP, mg/L (IQR)	18.4 (7.4–51.7)
Admission BNP (nmol/L) (IQR)	33 (15–82)
Admission creatinine, mmol/L (IQR)	71 (61–83)
Admission hemoglobin, g/dL (IQR)	140 (130–150)
Leucocytes count at admission (IQR)	11.7 (9.2–14.4)
Total cholesterol, g/L (IQR)	2.01 (1.70–2.36)
LDL cholesterol, g/L (IQR)	1.26 (1.0–1.56)
HbA1c, % (IQR)	5.7 (5.4–6.2)

CRP, C-reactive protein; BNP, brain natriuretic peptide, LDL, low-density lipoprotein.

**Figure 1:**
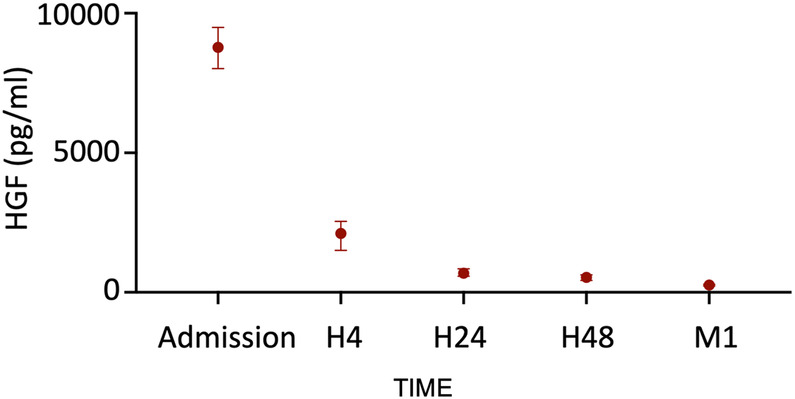
HGF release in our cohort of STEMI patients (*n* = 229). Median with interquartile.

To evaluate the kinetics of heparin-induced HGF release, we measured HGF levels at five different time points following STEMI by using ELISA. We observed a major peak of HGF at hospital admission (8,750 pg/mL, IQR: 8,021–9,492). The levels then decreased rapidly within the first 4 h to reach a relatively stable level from H24 to 1 month (Figure 1). The HGF levels did not correlate with ischemia time, age, or creatinine levels at admission. In addition, the levels were comparable between males and females.

**Figure 2:**
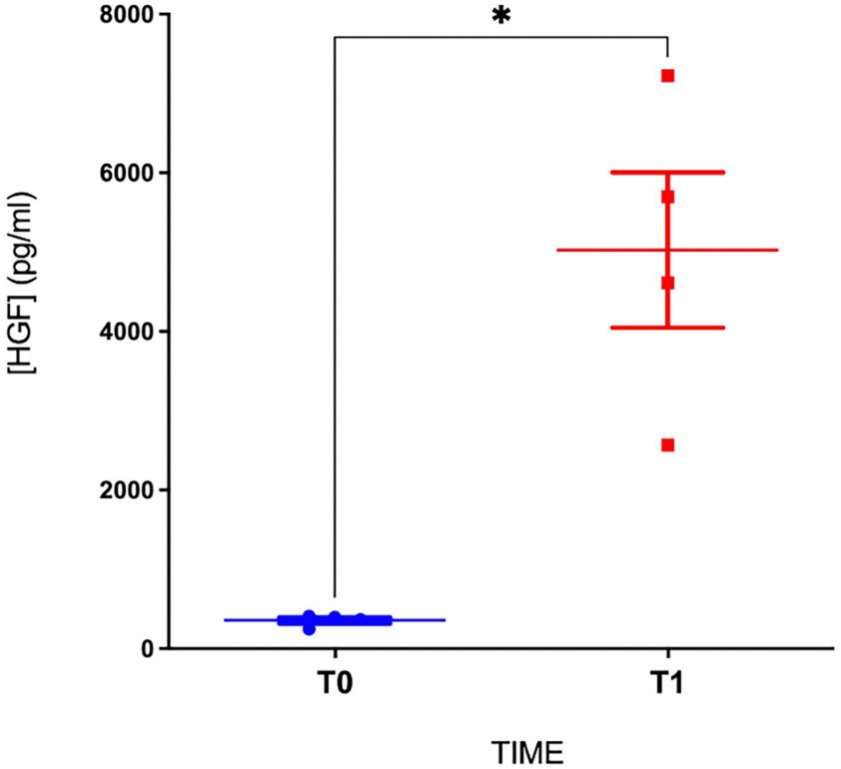
HGF levels before and after the bolus of heparin. T0, before heparin injection; T1, 30 min after the bolus of heparin (*p < 0.05; *n* = 4 patients).

To assess the impact of heparin injection on HGF levels, we compared the levels in four patients from the cohort before and 30 min after the administration of heparin bolus, using blood samples collected before catheter insertion during transport to the hospital. We observed a significant increase in the HGF levels after heparin injection (from 356 ± 77 pg/mL at baseline to 5,026 ± 1,957 pg/mL at peak; *p* < 0.05, Figure 2), confirming that the observed HGF was indeed heparin-dependent.

**Figure 3:**
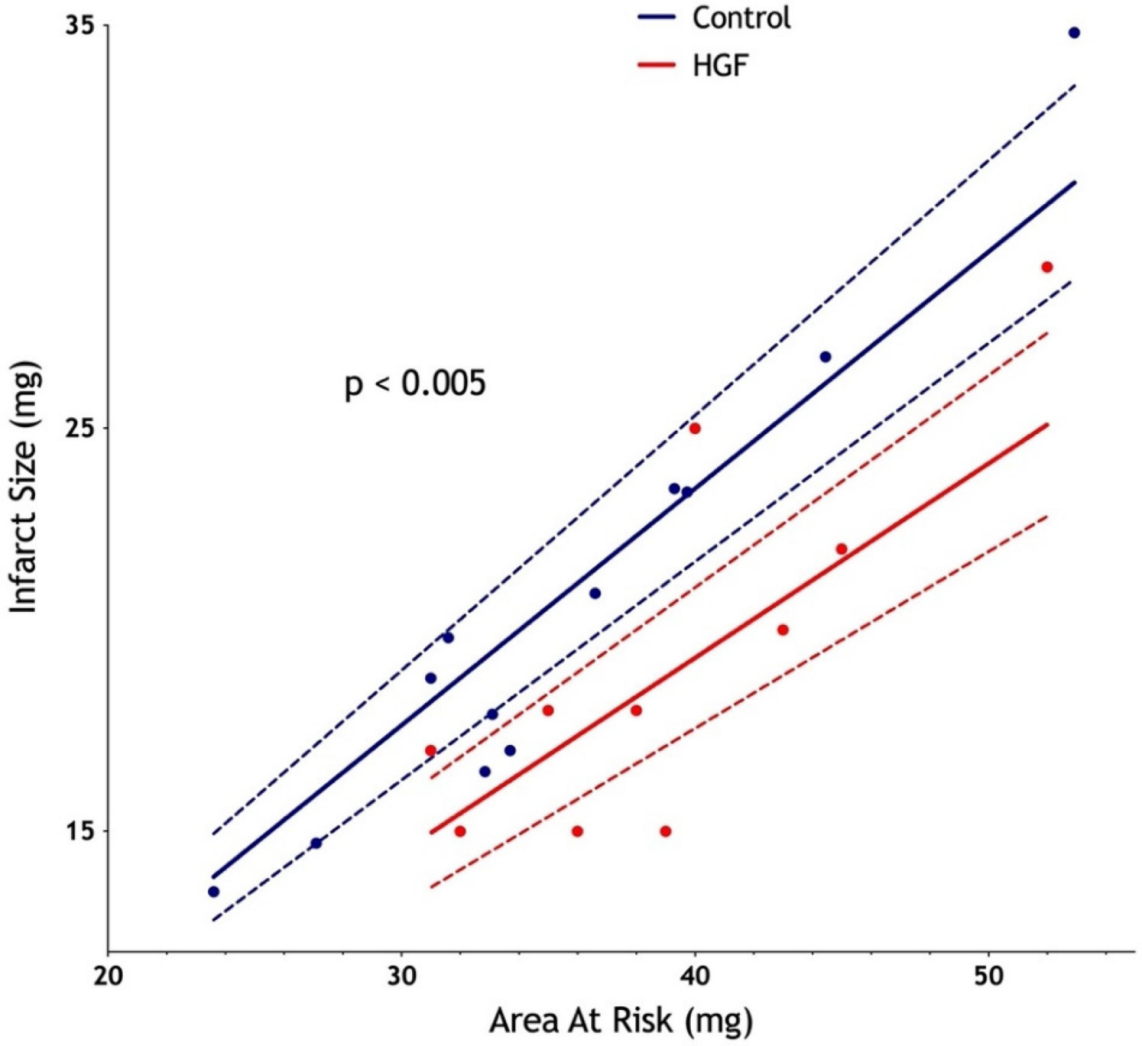
Significant diminution of infarct size after an intravenous injection of recombinant HGF 5 min before reperfusion in a murine ischemia-reperfusion model. Ischemia was initiated for 60 min, followed by a 24 h reperfusion. Infarct size was measured by the enzymatic technique (TTC, Unisperse blue dye). *n* = 12 in the control group; *n* = 11 in the HGF group.

To investigate the potential cardioprotective role of HGF in the early reperfusion in STEMI, we used a murine model of STEMI where recombinant HGF was injected 5 min before reperfusion time. The control group was constituted by the injection of physiological saline. After ischemia-reperfusion injury, we observed a similar area at risk between the two groups (median: 33.4 mg in the control group vs. 38.0 mg in the HGF group; *p* = 0.22), but a significantly reduced infarct size in the HGF-treated group compared with the control group (mean: 48% vs. 58%; *p* = 0.0023; 95% CI: 3.64–16.07%), which corresponded to a relative reduction of approximately 10% (Figure 3).

## Discussion

This translational study highlights the role of heparin in myocardial ischemia-reperfusion injuries and its potential impact on cardioprotection strategies.

Our study confirms a massive and extremely rapid release of HGF after intravenous injection of heparin in STEMI patients. Serum concentrations reached about 20 times the basal level, within the first few minutes. This is especially relevant for STEMI patients who are saturated with HGF during reperfusion in the cath lab. Using our preclinical model of STEMI*,* we observed a significant decrease in infarct size in the HGF group (48% vs. 58%, *p* < 0.005). These results suggest the potential cardioprotective role of HGF at the onset of reperfusion.

Although the indirect demonstration of heparin-induced HGF release is one of the limitations of this study, a direct administration of heparin was not possible due to its poor tolerance and the presence of risk of fatal hemorrhagic complications in animal models, which will render such experiments unethical (8). Nonetheless, the observed kinetic pattern strongly supports the role of heparin in triggering HGF release. In addition, the study pertains to the early stages of ischemia-reperfusion (the first 24 h), where many potentially cardioprotective molecules have been tested. We do not address the potential effects of HGF release on chronic remodeling.

Several clinical studies comparing heparin with bivalirudin during the acute phase of myocardial infarction have suggested a possible reduction in major cardiovascular events with heparin (9, 10). The HEAT-PPCI and EUROMAX trials, which evaluated anticoagulation strategies during primary percutaneous coronary intervention in STEMI patients, highlighted key differences in administration timing, access routes, and outcomes. In HEAT-PPCI, UFH was administered in-laboratory prior to angiography, whereas in EUROMAX, bivalirudin was initiated in a prehospital setting and compared with UFH or LMWH. Radial access was predominant in HEAT-PPCI (80%) vs. 48% in EUROMAX. Short-term outcomes showed higher rates of acute stent thrombosis with bivalirudin (HEAT-PPCI: 3.4% vs. 0.9%; EUROMAX: 1.1% vs. 0.2%) and a trend toward increased 30-day cardiovascular mortality in HEAT-PPCI, mainly because of reinfarction, cardiogenic shock, or fatal arrhythmias. EUROMAX demonstrated reduced major bleeding with bivalirudin (2.6% vs. 6.0%) and a trend toward lower 30-day mortality (2.1% vs. 3.5%), although the difference in early mortality was not statistically significant. At 12 months, HEAT-PPCI showed higher all-cause mortality in the bivalirudin group of patients (10.5% vs. 7.2%), driven by late MI, heart failure, or sudden cardiac death. Although EUROMAX initially suggested a lower incidence of early adverse events with bivalirudin, this benefit was offset by more events between 30 days and 1 year, resulting in comparable long-term mortality between the groups. The release of HGF triggered by heparin could contribute to the interpretation of these results.

Our data highlight a potential role of the heparin-induced HGF peak during the acute phase of myocardial infarction. To our knowledge, the link between heparin, HGF, and cardioprotection in the acute phase of STEMI has not been previously reported. Further studies aimed at elucidating the underlying mechanisms would be of significant interest to the cardiovascular research community.

## Data Availability

The processed data supporting this study are publicly available for download at the Figshare repository: https://figshare.com/articles/dataset/HGF_kinetic_in_the_human_cohort/30187978?file=58157794.
